# Fascination

**Published:** 1844-11

**Authors:** 


*Fascination.*—The late Lord —— had a daughter, who falling ill at Bath, was attended by the late Mr. ——. Of course he soon persuaded her that she had a stricture of the rectum. The young lady wrote to her mama in town an account of her case, and of Mr. ——'s procedure to cure her. Mama communicated this to papa, who not liking the thing much, went straight to an eminent practitioner, who showed his Lordship the great improbability of his daughter being affected in the manner indicated; and made him aware that Mr. ——'s patients were all—men or woman, matron or maid—presumed to be affected with stricture of the rectum, and treated accordingly. His Lordship was in a great fume; he would not even write; he posted off the same evening to rescue his child from the malapraxis, as he believed it, of Mr. ——. But what ensued? The London surgeon received a letter in a couple of days from Lord ——, to say that his

Lordship felt his visit to have been quite a providential one; that Mr. —— had not only satisfied him that his daughter had a stricture of the rectum, but that he himself had one, and that he meant to remain at Bath to have it cured!

*Med. Gaz.*, from *Boston Med. and Surg. Jour.*

